# Diagnosis of leptospirosis in animals: challenges and perspectives

**DOI:** 10.1128/jcm.01700-24

**Published:** 2025-10-30

**Authors:** Myranda Gorman, Bryanna Fayne, Sreekumari Rajeev

**Affiliations:** 1 Department of Biomedical and Diagnostic Sciences, University of Tennessee College of Veterinary Medicine 70737, Knoxville, Tennessee, USA; 2 Comparative and Experimental Medicine Program, University of Tennessee College of Veterinary Medicine 70737, Knoxville, Tennessee, USA; Vanderbilt University Medical Center, Nashville, Tennessee, USA

**Keywords:** *Leptospira*, leptospirosis, diagnostics, veterinary microbiology

## Abstract

Leptospirosis is a life-threatening zoonotic disease with a major public and animal health impact. Annually, approximately 1.03 million people are affected by leptospirosis, but our understanding of its impact on animals is limited. The epidemiology, pathogenesis, clinical presentation, diagnostic methods, and control measures for this disease differ significantly between humans and animals. This difference is due in part to the wide range of animal species that can be infected, the different infecting serovars present across species and geographic regions, and the existence of chronic asymptomatic reservoirs. Additionally, diagnosing leptospirosis in animals is complicated by the limited availability of sensitive, specific, and affordable diagnostic tools that can be employed at the point of care. There is often a trade-off between the sensitivity/specificity and accessibility of these diagnostics, and no single diagnostic test is entirely reliable. Many newer diagnostic methods lack validation for use in various animal species and clinical samples. In this minireview, we discuss the methods used for detecting *Leptospira-*infected animals and challenges associated with these techniques.

## INTRODUCTION

Leptospirosis is a global, neglected zoonotic disease that impacts both human and animal health. Leptospirosis is caused by the pathogenic members of the spirochete bacterial genus *Leptospira* (1, 2). Pathogenic *Leptospira* colonizes the proximal renal tubules of numerous mammalian species, thus facilitating its excretion through urine, resulting in transmission to susceptible hosts or environmental contamination. A large variety of pathogenic and saprophytic *Leptospira* persist in the environment, and contact with the contaminated environment can be a major mode of transmission (3). Global annual human leptospirosis cases and associated deaths are estimated at 1.03 million and 60,000, respectively (4). Nevertheless, estimates of its deleterious impact on animal populations are not available. This is largely due to its wider impact on a large variety of animal species, of which some may develop serious life-threatening diseases, while others remain asymptomatic reservoirs. In addition, epidemiology, pathogenesis, clinical presentation, diagnostic methods, and control measures in animals differ from humans. Acute clinical symptoms are documented in a variety of animal species, but there is also chronic infection that can result in maintenance and continuous shedding of the bacteria (5).

The traditional nomenclature classifies *Leptospira* into serovars based on their serologic reactivity, and cross-reactive serovars are categorized into serogroups (1, 2). The traditional classification methods employed cross-adsorption agglutination assays and monoclonal antibody-based methods that are cumbersome and only available in reference laboratories (6). Currently, the genus *Leptospira* is composed of at least 64 species based on genetic sequences that are classified into pathogenic and saprophytic clades (2).

*Leptospira* infection in animals can lead to a wide range of presentations depending on the infecting serovar, the animal host infected, and the immune status of the host. The pathogenic mechanisms are complex and poorly understood. Acute clinical cases present as febrile disease with broad manifestations resembling other infections and then progress to icteric and anicteric presentations (7–10). Similar to humans, untreated cases present a biphasic pattern where *Leptospira* disseminates through the blood in the initial stages, followed by renal colonization and an immune phase (1, 6). Diagnosis of chronic and subclinical *Leptospira* infection is challenging due to the absence of compatible clinical signs. For example, ruminants that develop chronic *Leptospira* infection will have renal or genital colonization and intermittent shedding without significant clinical disease (11, 12). Diagnosis of *Leptospira* infection in animals is generally based on the basic principles of infectious disease diagnosis: the detection of the organism or its components and the evaluation of host response following exposure. In this review, we discuss the methods used for the detection of *Leptospira* and host response in animals and the challenges associated with using these methods.

## CLINICAL PRESENTATION OF *LEPTOSPIRA* INFECTION IN VARIOUS ANIMAL SPECIES

Animal species generally present with a range of broad clinical manifestations. Most acute clinical cases present as febrile disease during the initial bacteremia phase, which goes largely undetected in many animals due to non-specific manifestations. Animals presenting with clinical pathology abnormalities, including markers of hepatic and renal dysfunction, should be considered as suspect cases (7, 8, 13). However, not all species will present with notable hepatic or renal dysfunction. In ruminants, reproductive failure is one of the major manifestations of chronic colonization (14). In horses, in addition to systemic disease, chronic colonization of the eyes can lead to recurrent equine uveitis and blindness (15).

## *LEPTOSPIRA* DETECTION METHODS

### Microscopy

**Fig 1:**
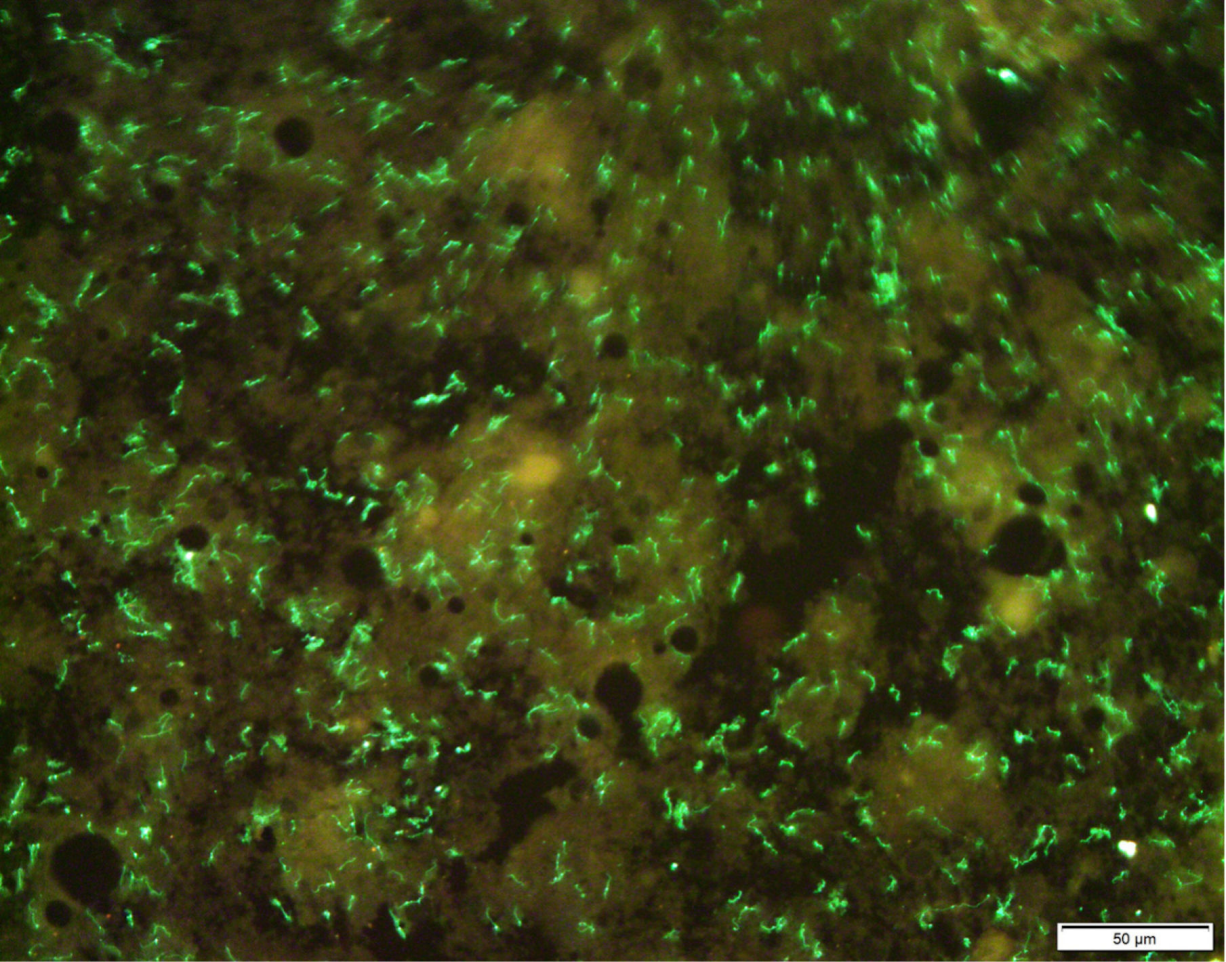
Immunofluorescence assay for the detection of *Leptospira* in the kidney of a rat.

*Leptospira* are thin spirochete bacteria and cannot be visualized by brightfield microscopy or routine Gram staining. However, the ability of some of these stains to presumptively detect *Leptospira* cannot be ignored. A recent study reported the presence of suspect organisms in the urine sediment of a dog with clinical leptospirosis using routine cytology staining (16). The spiral shape and peculiar motility allow for their detection using a darkfield microscope (DFM) in clinical specimens such as body fluids when *Leptospira* are in abundance (17, 18). The test has low sensitivity, specificity, and many laboratories are not equipped with darkfield microscopy (19). Despite being a rapid presumptive method, the presence of contaminants, such as saprophytic *Leptospira* and other spirochete bacteria with similar morphology that are ubiquitous in water and the environment, can lead to misdiagnosis. Clinical samples may contain fibrin and other tissue debris that mimic the *Leptospira* morphology as well, leading to misdiagnosis when evaluated by a technologist with inadequate training. Silver stains, such as Warthin-Starry, are utilized to aid in the visualization of organisms in formalin-fixed tissue samples (20, 21). Silver stains are not specific to *Leptospira* and can also stain other spirochete bacteria and argyrophilic membranes in the tissue, thereby reducing their specificity. Immunofluorescence assay using a FITC-conjugated polyclonal anti-*Leptospira* antibody can identify the presence of *Leptospira* in clinical samples, such as urine and tissue (22, 23). The presence of fluorescent organisms with spirochete morphology can be easily distinguished (Fig. 1). However, the requirement of a fluorescent microscope, an expensive piece of equipment, limits the number of laboratories that can perform this test.

## *LEPTOSPIRA* CULTURE

**Fig 2:**
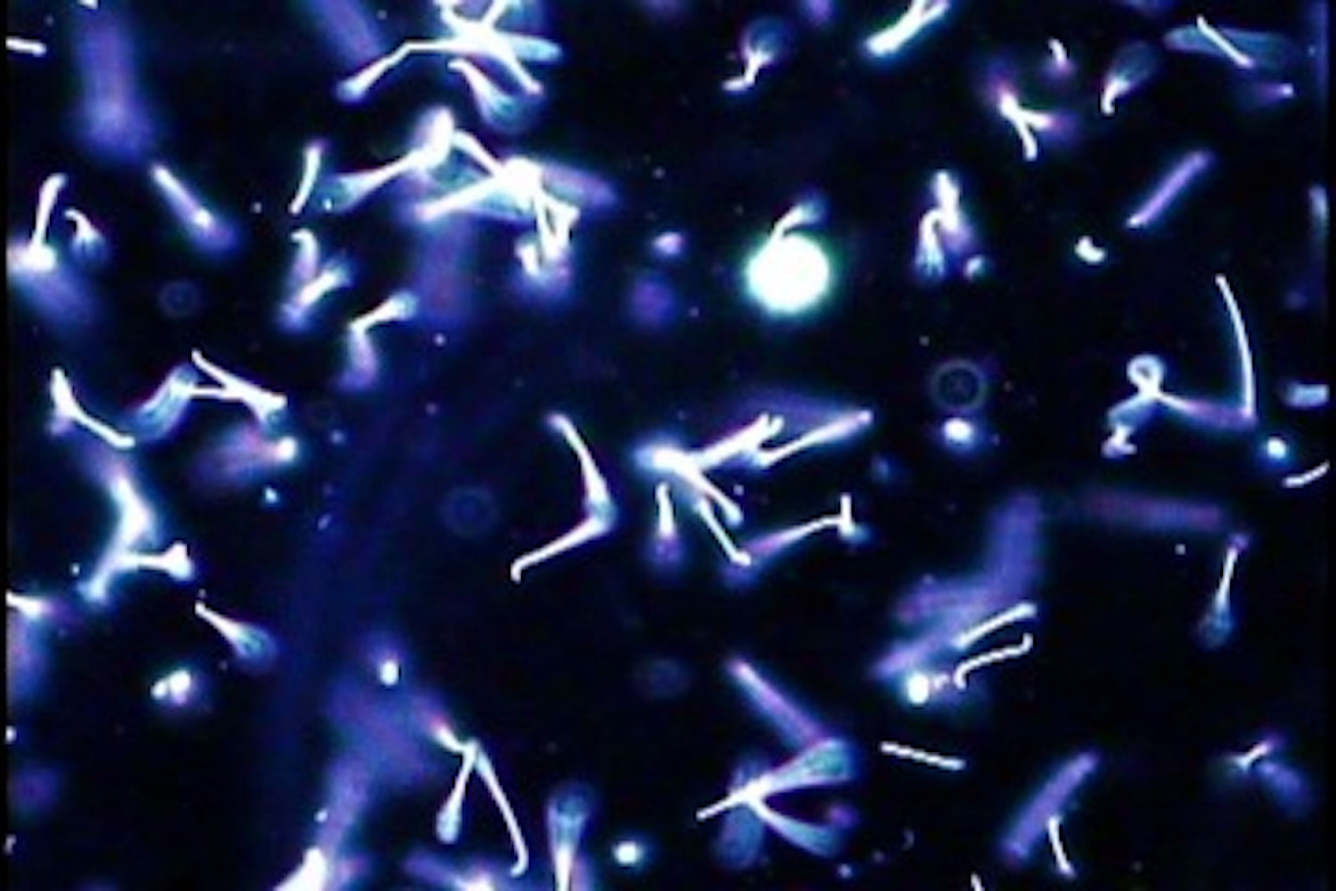
Darkfield microscopy of *Leptospira* culture.

Although isolation and identification of pathogens from an infected host are always considered the gold standard, this approach may not be meaningful in cases of infections caused by fastidious bacteria such as *Leptospira* because of their unique growth requirements and prolonged incubation period (24, 25). The initial isolations of *Leptospira* were performed using peptone and beef extract-containing media, such as Fletcher’s, Stuart, and Korthoff’s media (19, 26–28). Later, a serum-free medium, Ellinghausen-McCullough-Johnson-Harris (EMJH) media, was developed and is one of the most used laboratory media for culturing *Leptospira*. However, some of the *Leptospira* strains are fastidious and grow better with the addition of enrichments such as rabbit sera, fetal bovine serum, lactalbumin hydrolysate, superoxide dismutase, B12, Tween 80, glycerol, and sodium pyruvate (29–31). In recent years, a new media, HAN, using Dulbecco’s Modified Eagle base, Ham’s Nutrient mixture F12, vaccine-grade bovine serum albumin, and other chemicals required for optimal *Leptospira* growth, was developed (32). The HAN media was found to be superior in isolating fastidious *L. borgpetersenii* strains at the routine 29°C and 37°C incubation temperatures. Comparison of HAN media to T80/40/LH media showed that, overall, both media performed similarly; however, HAN was generally able to culture *Leptospira* quicker than T80/40/LH. One of the drawbacks is the cost of the components recommended, especially in resource-limited settings and when large-scale sample testing is required. *Leptospira* has a generation time of 6 to 20 h, with a few pathogenic species, such as *L. borgpetersenii*, having longer generation intervals requiring an increased incubation period (17, 33). Due to the prolonged incubation requirements, growth of contaminant bacteria can be a major issue during culture, so supplementation of antimicrobial agents is used frequently while isolating *Leptospira* from clinical samples (34). Traditionally, 5-fluorouracil has been used as an agent to prevent contamination and is still widely used (35). The addition of selective antibiotic cocktails, such as STAFF, has shown improved ability to prevent contamination while sustaining *Leptospira* growth (34, 36). In a recent study, we found that selective enrichment of samples with antimicrobials improved *Leptospira* detection from highly contaminated environmental samples (37). It must be noted that STAFF can slow down the growth and replication of *Leptospira* in culture, which often leads to no recovery of isolates. Sample handling and processing can also have a significant impact on isolating *Leptospira* from clinical samples. Samples collected from animals already treated with antibiotics are unlikely to have successful isolation of *Leptospira*. In many cases, fresh samples are preferred for isolating *Leptospira* because conditions in urine and blood samples lead to degradation, rendering *Leptospira* unculturable (38). Generally, 10^7^ or 10^8^ *Leptospira* are needed for a culture to appear grossly turbid, so frequent DFM examination during a prolonged incubation period is needed to confirm the bacterial growth (19). The presence of *Leptospira* in cultures can be confirmed by observing unique motility and morphology (Fig. 2), followed by confirmation of pathogenic *Leptospira* using PCR. Culture is typically not used for the diagnosis of clinical leptospirosis cases as it will not provide any critical diagnostic value. In addition, maintaining *Leptospira* cultures in the laboratory for further characterization is a labor-intensive and difficult task. However, isolation and maintenance of bacteria are crucial for epidemiological studies, research, and the development of vaccines and diagnostics.

## MOLECULAR DIAGNOSTICS

As discussed earlier, the traditional methods used for *Leptospira* detection from clinical samples have varying sensitivity and specificity, and unlike other bacteria, culturing is not useful for clinical diagnosis and timely intervention. Therefore, nucleic acid amplification tests (NAAT) have been widely used for the detection of infection in animal species. Higher sensitivity and specificity offer earlier, specific detection and intervention compared to other methods, including serology that relies on antibodies that generally appear around days 5 to 10 post-infection. Several types of PCR assays targeting multiple gene targets, including *lipL32*, *secY*, *lfb1*, *rrs*, and *flaB*, have been evaluated, with *lipL32* being the most utilized (39–43). *The lipL32* gene target is widely used and has high specificity for detecting pathogenic *Leptospira,* and a significant amount of literature has been published on its performance with different sample types, such as blood and urine. The sensitivity and specificity of these assays can vary depending on the gene targets and samples tested. *SecY* is a common target used in various studies but lacks definitive specificity for pathogenic *Leptospira* (44, 45). While it has limitations as a diagnostic target, its sequence variability makes this gene target suitable for further characterization of isolates (45). Despite its use in identifying isolates, some reports suggest that *secY* may not amplify clinical isolates reliably, highlighting the need for multiple gene targets (45–47). In our lab, we routinely retest *lipL32-negative* clinical samples using the *rrs-based* PCR (48), occasionally uncovering some false-negative results (data not shown). Because of a large repertoire of *Leptospira* strains circulating in the environment and, unlike humans, the diversity of animal species that can be exposed to these strains, careful selection and validation of gene targets are essential for accurate results. PCR inhibitors are a common problem reducing detection sensitivity, which can be overcome by the use of improved DNA extraction kits and PCR reagents, along with inclusion of internal quality controls (49–51). Nested PCR has been suggested as an alternative PCR strategy to improve the sensitivity and specificity of conventional PCR (52, 53). However, the chance of contamination and false positives is a serious concern (54). One of the most common *lipL32*-based real-time PCR assays widely used for *Leptospira* detection has undergone modifications to achieve higher sensitivity and specificity (55, 56). A promising culture-independent NAAT was recently reported (*RAg1*) that reliably detects and identifies *Leptospira* species and serovars (57). Sample types tested in animal species are numerous and varied, and thus, appropriate selection and processing of samples is critical to avoid false-positive and negative results.

The recently introduced digital PCR method offers several benefits, including fewer false negatives arising from PCR inhibitors, accurate quantification of bacteria without the necessity of running standard curves, and an impressive sensitivity that allows for the detection of small amounts of DNA in samples (58). An RNA-based digital PCR targeting a flagellar motor switch protein gene, *fliG*, has been shown to have higher sensitivity compared to *lipL32* and has been successfully used to detect low-level *Leptospira* (59). The cost of equipment and reagents remains a major limiting factor in introducing this technology to resource-limited areas. Lack of standardization for digital PCR testing of *Leptospira* also limits its current implementation in a diagnostic setting and highlights the need for further optimization of digital PCR testing.

One of the major barriers to *Leptospira* detection is the lack of affordable and sensitive point-of-care or field tests to identify infected animals, especially in resource-limited areas. Rapid amplification methods with shorter turnaround that do not require expensive equipment or skilled personnel, such as loop-mediated isothermal amplification (LAMP) assays and recombinase polymerase amplification (RPA), have been explored for *Leptospira* detection (60–64). Advances such as the use of Bst polymerase in LAMP have overcome the commonly observed effect of amplification inhibitors in complex biological samples to some extent (65). The endpoint detection method is critical to improving sensitivity. Many modifications have been added, including fluorescence and colorimetric detection for interpretation; although useful, these are at the cost of reduced specificity (66, 67). However, incorporation of gel electrophoresis or lateral flow assays enhances the specificity at the cost of requiring additional laboratory equipment and skills (68, 69). The integration of several CRISPR/Cas-based detection platforms, including DETECTR, HOLMES, and SHERLOCK, has enabled higher detection specificity and sensitivity (70–72). In comparison to LAMP, RPA is a better candidate for testing samples in resource-limited areas. RPA reactions can be incubated using body heat and have a quicker turnaround time (64, 73). The implementation of RPA in the diagnosis of leptospirosis is limited by sparse research on clinical testing for pathogenic *Leptospira* and limited commercial options in comparison to LAMP. Aptamer-based diagnostic methods are another diagnostic tool gaining popularity due to their ability to directly capture target DNA or RNA from samples without nucleic acid extraction. Two recent papers investigated the utility of aptamer assays targeting *lipL32* to detect *Leptospira* in human clinical samples (74, 75). One of the biggest obstacles to implementation in animal testing is the requirement for validation across various animal species and sample types.

## CHARACTERIZATION OF *LEPTOSPIRA* ISOLATES

The strain diversity of *Leptospira* in animals and the environment is vast and largely unknown, and there is geographic variation in their prevalence. Identifying the species as well as the serovar of *Leptospira* isolates is vital to study the epidemiology and transmission of leptospirosis and guide the selection of antigens for vaccines. Identifying *Leptospira* at the serovar level is useful for vaccine and diagnostic development. Rabbit polyclonal anti-*Leptospira* antibodies can be used to characterize the isolates at the serogroup level. However, definitive serovar identification requires the cross-agglutination adsorption assay and monoclonal antibody-based assay, which are laborious, expensive, time-consuming, and available only in some reference laboratories. DNA-based methods, such as multilocus sequence typing (MLST), have been developed to identify genotype/strain type (76–78). Whole-genome sequencing has become a common tool for characterizing *Leptospira* isolates and is the gold standard for species- and strain-level classification to study genomic diversity and evolutionary relationships (2). More sophisticated approaches using long-read and short-read sequencing technology and hybrid assembly have uncovered a more detailed characterization of *Leptospira* isolates (79). Analysis of loci such as the *rfb* locus, which codes for lipopolysaccharide biosynthesis, has recently been explored for its ability to differentiate between serovars (80, 81). Due to the complexity and variation of this locus resulting from horizontal gene transfer, and the limited number of complete sequences of this locus in public databases, optimization of these techniques has been a daunting task (82). A recent paper explored the usage of PCR primers based on the *rfb* locus for serovar classification of *Leptospira*. However, the primers were only able to classify at the serogroup level (83). Matrix-assisted laser desorption/ionization time-of-flight mass spectrometry (MALDI-TOF) is another promising technology to type bacterial species (84). A handful of research papers have suggested that MALDI-TOF could be used as a rapid diagnostic to determine the species of infective *Leptospira* (85–87). However, a clinical isolate is required for MALDI-TOF classification. Isolation of *Leptospira* is not routinely achieved from clinical samples, which makes this procedure rarely achievable.

## METAGENOMIC SEQUENCING

With advances in the next-generation sequencing methods, it is now possible to comprehensively and effortlessly sequence and identify the total nucleic acid content of a clinical sample to identify significant pathogens. A couple of reports have shown the significance of this method in detecting *Leptospira* in human patients when other common diagnostic tests, such as MAT and PCR, failed (88–91). Although very useful in identifying the infectious etiology, insufficient target DNA, as well as the cost and limited availability of sequencing, analysis, and interpretation services, are major drawbacks. To improve detection, use of capture probes followed by sequencing has grown in popularity as a culture-free form of *Leptospira* isolation from animal samples (92–94). Usage of capture probes eliminates the need for time-consuming isolation of *Leptospira* from clinical samples to further characterize the infecting *Leptospira* strain (94). However, high cost and increased technical expertise limit the adoption of capture probes for routine diagnosis and investigation (92). Our lab has successfully used enrichment culture followed by metagenomic sequencing to identify the diversity of *Leptospira* in the environment, and the same can be applied to clinical samples for identifying and characterizing *Leptospira* strains without compromising the sensitivity (37).

## DETECTION OF HOST RESPONSE

The microscopic agglutination test (MAT) is one of the oldest and routinely used serologic methods for detecting anti-*Leptospira* antibodies (95). MAT is run using a panel of live cultures of *Leptospira* serovars maintained in the laboratory through continuous passage. Antibodies against *Leptospira* lipopolysaccharide and outer membrane, if present in the serum samples tested, will agglutinate the bacteria. Then, the reaction can be visualized by DFM. Although considered the gold standard reference serologic test, its utility in clinical diagnosis of leptospirosis is limited due to multiple factors. Unlike humans, many animal species are exposed to *Leptospira* and asymptomatically harbor the bacteria in the renal tubules and occasionally in the reproductive tract, which may not induce an agglutinating antibody response. Some of the reservoir hosts, such as rodents and cattle, while actively infected, can be MAT negative (23, 96). For instance, in a rodent prevalence study conducted in an endemic area, we observed rats infected with *L. interrogans* strains were positive for antibodies by MAT, but those infected by *L. borgpetersenii* strains were negative (96). Rats with mixed infections with both strains showed elevated antibody response to only *L. interrogans*.

Acutely ill animal patients may also test MAT negative. Therefore, relying on MAT alone may lead to false-positive or false-negative results. In human studies, it has been shown that the true sensitivity of even a combination of reference tests, such as MAT and *Leptospira* culture, is low and may lead to misdiagnosis (97). In addition, paired titers using acute and convalescent sera from patients are recommended for a definitive diagnosis, although the immediate clinical benefit of managing the disease is questionable with this approach (98, 99). MAT results are limited to the serovars included in the panel. It is important to curate the serovars present on the MAT panel due to the regional variation in prevailing serovars (100–102). A 1.9% to 16.9% increase in prevalence in rats was reported when tested with a MAT panel that included locally isolated serovars (103). In vaccinated animals, MAT titers may be elevated for serovars present in vaccines (104). A high titer to a specific serovar cannot be trusted implicitly as the infecting serovar, since paradoxical titers (positive titers for unrelated serovars) are common in early stages of clinical disease (6). In humans, a MAT titer of 800 is considered a positive case. In animals, reservoir and vaccination status can confound the MAT titers, and hence, a specific titer should not be recommended for determining positive infection status. Instead, adjustments in cutoff titers should be made based on endemicity, animal species, and geographic region (98). Identifying antibody prevalence to specific serovars has epidemiological merit since some serovars are host-adapted and may provide clues to prevailing serovars. Often, interpretation of MAT results is difficult due to the cross-reactivity between serovars, which is common in animals (105). A recent study emphasized that the highest antibody titer is not a reliable indicator of the infecting serovar and discussed the role of host species in shaping reactivity patterns (106). As explained, seronegativity against a given serovar does not rule out *Leptospira* as a cause of infection. Other factors, such as host species exposure, vaccination status, and variation between testing in diagnostic laboratories, should be considered in the system under surveillance. Technical factors to consider are biosafety concerns of handling live organisms and difficulty in laboratory standardization of the MAT test, leading to variation in results between laboratories (6, 101). Replacing MAT with another appropriate and less complex serologic test has been a challenging issue. Unlike humans, only a few rapid diagnostic tests are available for anti-*Leptospira* antibody detection and are useful for early presumptive diagnosis of leptospirosis and disease management. A test targeting antibodies against *lipL32*, an abundant membrane protein of pathogenic *Leptospira*, as well as an IgM detection against *Leptospira* whole-cell extract, is available for dogs. The IgM-based detection test identified antibodies to *Leptospira* earlier than the MAT test when tested in experimentally infected dogs. Comparison of the *lipL32* assay with MAT results found that there was 83.2% agreement between this ELISA and MAT results (107). Similar to MAT, these tests can also be impacted by recent vaccination (108).

Commercially available ELISAs for the diagnosis of leptospirosis in animals are not common. Currently, no antigen detection assays are commercially available, although there are a handful of studies investigating this as a potential diagnostic (109, 110). A few antibody capture ELISAs have been developed, typically involving key *Leptospira* antigens. One group developed a bovine leptospirosis ELISA with recombinantly expressed *lipL32* as the antigen that had sensitivity and specificity of 100% in relation to positive MAT samples with a titer of 1:100 or greater (111). Additionally, another group using *lipL32* as their key antigen reported 95% specificity for an ELISA detecting positive samples in field rats (112). A variety of *Leptospira* antigens, such as *lipL32*, *lipL21*, *ompL1*, and more, have been investigated for diagnostic and research use in species, such as canine, bovine, equine, swine, and rats, with specificity and sensitivity averaging about 80% or higher (112–118). The motivation for developing these tests is to find a diagnostic that requires less laboratory expertise and maintenance in comparison to MAT. However, a setback for developing an in-lab ELISA for diagnostic use is properly validating the test and making sure the antigen used will properly detect positive and negative samples. An ELISA for the detection of *Leptospira* in field rats showed an 83% sensitivity for the *lipL32* antigen recombinantly expressed in *Pichia pastoris*, but only 50% sensitivity when the same antigen was expressed in *Escherichia coli*, potentially due to *E. coli* being a common environmental pathogen (112). A study comparing a point-of-care rapid test with MAT and another commercial test with samples from 32 dogs infected with the *Leptospira* serovars Canicola, Grippotyphosa, Icterohaemorrhagiae, and Pomona showed the detection of seroconversion in all infected dogs by day 10, whereas MAT detected only 30 infected dogs by day 17 (119). The lateral flow assays for other animal species, such as bovine, have been tested, but the commercially available tests remain limited (120). Lateral flow assays are useful for their extended shelf life, straightforward procedure, and clear results. However, the above-mentioned limitations to antibody-based diagnostics prevent their use as a singular diagnostic tool for animal leptospirosis.

## NECROPSY AND HISTOPATHOLOGY

**Fig 3:**
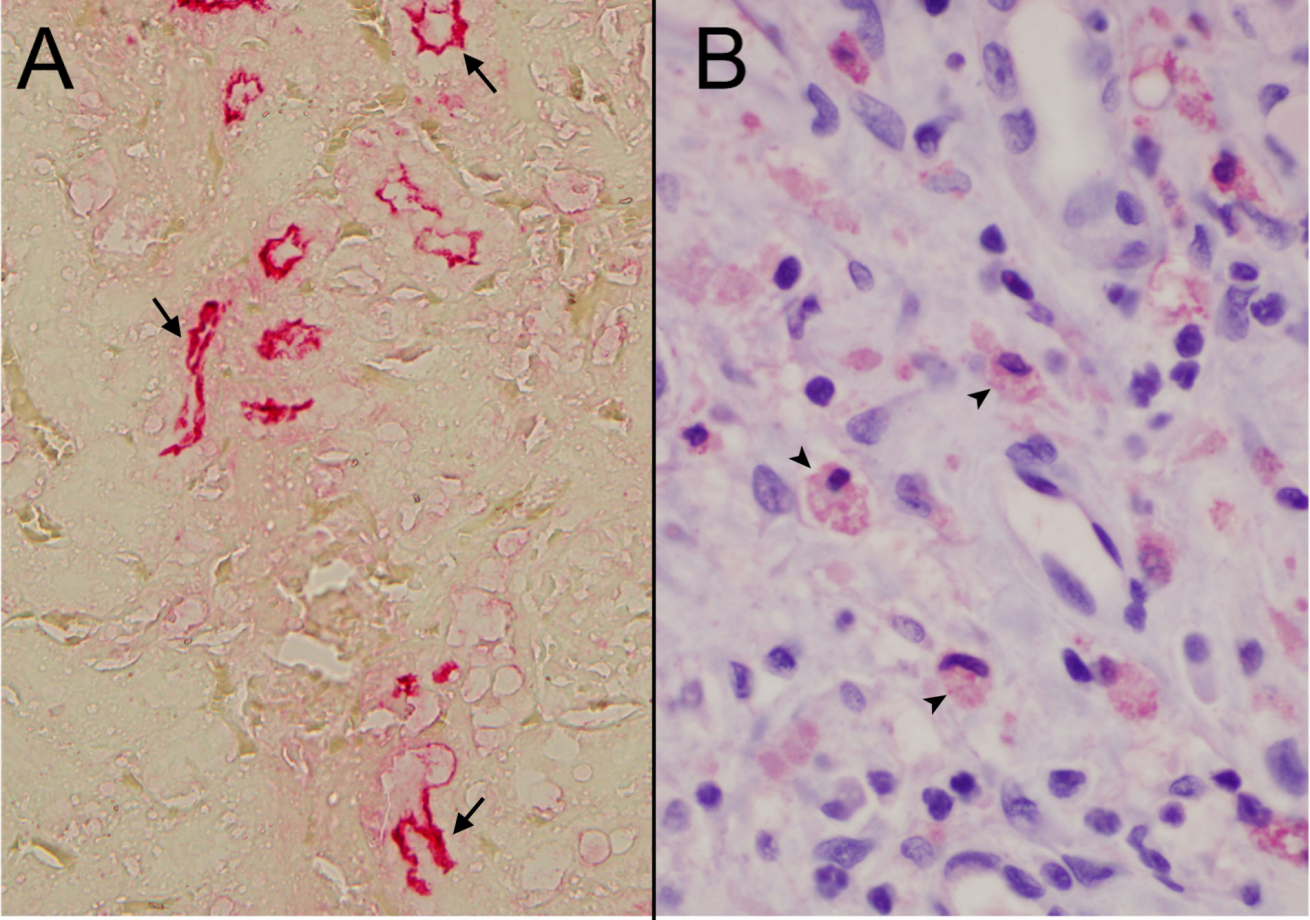
Immunohistochemistry (IHC) performed on formalin-fixed paraffin-embedded kidney sections for *Leptospira* antigen staining.* (**A**) Kidney from a rat (note the multifocal staining within the tubular lumen, pointed by arrows). (**B**) Kidney from a canine clinical leptospirosis case (note the diffuse antigen presence mostly within the macrophages pointed by arrow heads). Both images are from IHC but were performed in two different laboratories at different times.

Postmortem examination followed by histopathology can be an ideal tool to rule out leptospirosis as a cause of death. Cases can present as anicteric or icteric forms of disease. In icteric forms, jaundice and liver abnormalities are the major findings, and in anicteric forms, renal abnormalities predominate. In some cases, changes in both organs can be observed. Histopathologic changes observed in the liver are mostly hepatocellular dissociation without associated inflammation (7), whereas in the kidney, a variety of changes, including renal tubular necrosis and interstitial nephritis, can be seen (8). Suspicious cases can be confirmed by PCR or immunohistochemistry. Immunohistochemistry, another antigen detection method, is performed on formalin-fixed histological specimens, such as the liver and kidney of infected animals. Utilizing this stain, *Leptospira* antigens can be visualized with light microscopy (Fig. 3). A diffuse staining pattern and associated inflammation can be observed in acute clinical cases, and a more multifocal reactivity within the renal tubules is seen in chronic reservoir animals. However, an optimized antigen retrieval procedure to unmask antigens for effective antibody binding is crucial for this procedure.

## SUMMARY

The epidemiology, pathogenesis, clinical presentation, diagnostic approaches, and control measures for *Leptospira* infection vary widely due to the broad range of susceptible animal species and the numerous serovars circulating across diverse geographic regions. Although several diagnostic methods are available to identify *Leptospira*-infected animals, each has limitations related to sensitivity, specificity, cost, availability, and the need for specialized expertise. A major challenge in this field is the limited availability of sensitive, specific, and affordable point-of-care diagnostic tools—particularly in resource-limited settings. This issue is especially concerning for subsistence farmers who rely heavily on animal health for their livelihoods. The need to employ multiple diagnostic modalities further complicates detection, as no single test offers complete reliability. The diversity of affected animal species, the geographic variation in circulating *Leptospira* strains, and the presence of chronic asymptomatic carriers hinder the development and implementation of standardized diagnostic guidelines. While traditional methods may be useful in certain contexts, they often lack adequate sensitivity and specificity. Serologic tests, though commonly used, can be difficult to interpret in animals, necessitating confirmatory testing in positive cases.

Molecular diagnostics, such as PCR, offer enhanced sensitivity and specificity depending on the gene target and sample type. However, clinical signs of leptospirosis in animals are often nonspecific and may mimic other diseases during the acute phase. Therefore, a syndromic testing approach is recommended to facilitate early intervention.

**Fig 4:**
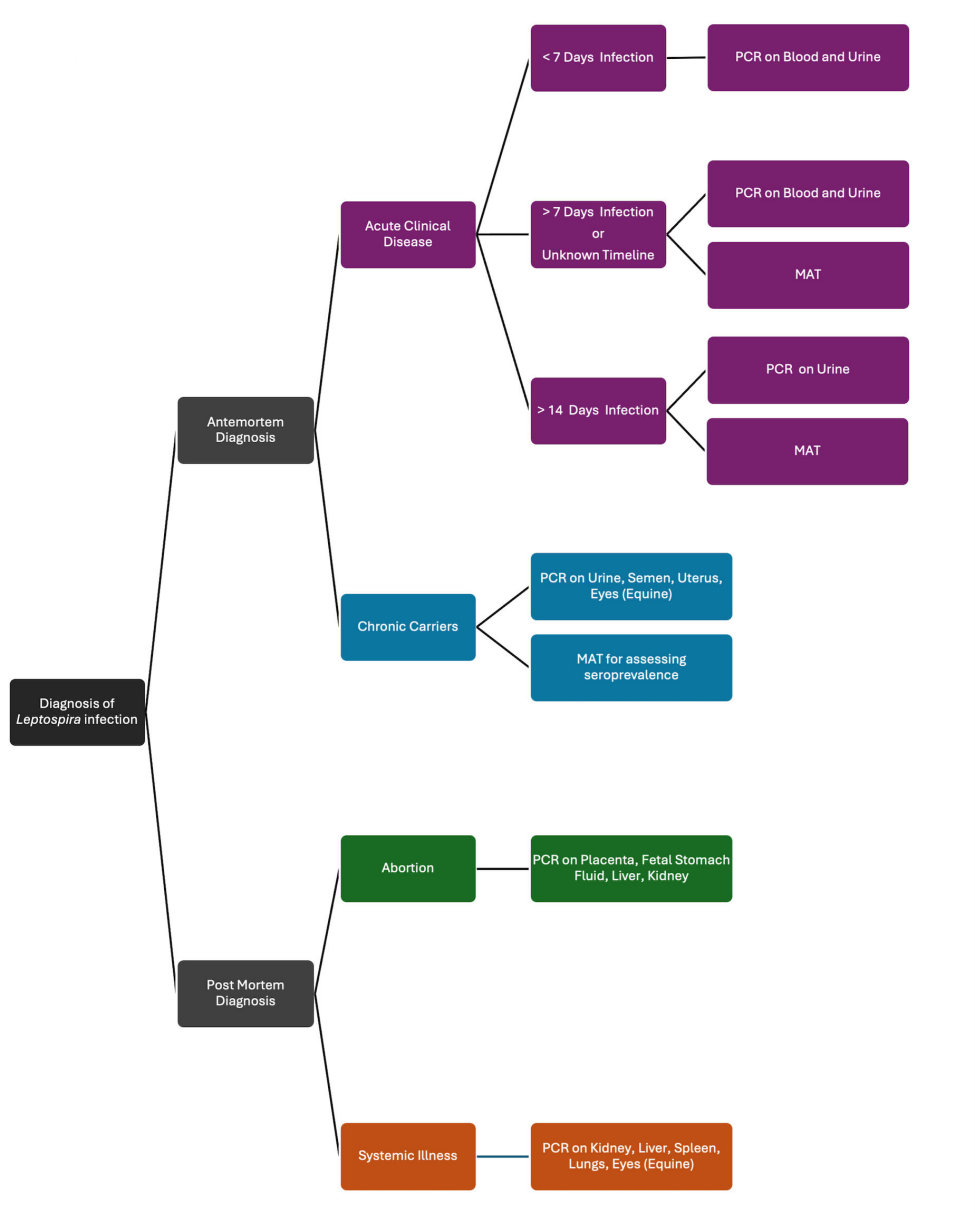
Guidelines for targeted testing of animals for *Leptospira* infection.

Given these challenges, Fig. 4 illustrates a recommended strategy for sample collection and diagnostic testing in both clinical and chronically infected cases. While a wide array of diagnostic technologies is available through diagnostic laboratories, there remains an urgent need for more accessible and cost-effective tools to support disease detection in regions where leptospirosis is most prevalent.
